# Comparison of two threshold detection criteria methodologies for determination of probe positivity for intraoperative *in situ* identification of presumed abnormal ^18^F-FDG-avid tissue sites during radioguided oncologic surgery

**DOI:** 10.1186/1471-2407-14-667

**Published:** 2014-09-13

**Authors:** Gregg J Chapman, Stephen P Povoski, Nathan C Hall, Douglas A Murrey, Robert Lee, Edward W Martin

**Affiliations:** Department of Electrical and Computer Engineering, The Ohio State University, Columbus, OH 43210 USA; Division of Surgical Oncology, Department of Surgery, Arthur G. James Cancer Hospital and Richard J. Solove Research Institute and Comprehensive Cancer Center, The Ohio State University Wexner Medical Center, Columbus, OH 43210 USA; Division of Molecular Imaging and Nuclear Medicine, Department of Radiology, The Ohio State University Wexner Medical Center, Columbus, OH 43210 USA

**Keywords:** ^18^F-fluorodeoxyglucose, ^18^F-FDG, Target-to-background ratio, Tumor-to-background ratio, Three-sigma, Ratiometric, Threshold, ^18^F-FDG-directed surgery, Radioguided surgery, Intraoperative detection, Gamma detection probes, *in situ*, Count rate, Neoplasms, Oncologic

## Abstract

**Background:**

Intraoperative *in situ* identification of ^18^F-FDG-avid tissue sites during radioguided oncologic surgery remains a significant challenge for surgeons. The purpose of our study was to evaluate the 1.5-to-1 ratiometric threshold criteria method versus the three-sigma statistical threshold criteria method for determination of gamma detection probe positivity for intraoperative *in situ* identification of presumed abnormal ^18^F-FDG-avid tissue sites in a manner that was independent of the specific type of gamma detection probe used.

**Methods:**

From among 52 patients undergoing appropriate *in situ* evaluation of presumed abnormal ^18^F-FDG-avid tissue sites during ^18^F-FDG-directed surgery using 6 available gamma detection probe systems, a total of 401 intraoperative gamma detection probe measurement sets of *in situ* counts per second measurements were cumulatively taken.

**Results:**

For the 401 intraoperative gamma detection probe measurement sets, probe positivity was successfully met by the 1.5-to-1 ratiometric threshold criteria method in 150/401 instances (37.4%) and by the three-sigma statistical threshold criteria method in 259/401 instances (64.6%) (P < 0.001). Likewise, the three-sigma statistical threshold criteria method detected true positive results at target-to-background ratios much lower than the 1.5-to-1 target-to-background ratio of the 1.5-to-1 ratiometric threshold criteria method.

**Conclusions:**

The three-sigma statistical threshold criteria method was significantly better than the 1.5-to-1 ratiometric threshold criteria method for determination of gamma detection probe positivity for intraoperative *in situ* detection of presumed abnormal ^18^F-FDG-avid tissue sites during radioguided oncologic surgery. This finding may be extremely important for reshaping the ongoing and future research and development of gamma detection probe systems that are necessary for optimizing the *in situ* detection of radioisotopes of higher-energy gamma photon emissions used during radioguided oncologic surgery.

## Background

The use of intraoperative gamma probe detection technology has become a mainstay in the surgical management of many solid malignancies [1]. While current commercially-available gamma detection probe technology works well for detecting radioisotopes of low-energy gamma photon emissions, such as ^99m^Tc (140 and 142 KeV), the detection of radioisotopes of higher-energy gamma photon emissions, such as ^18^F-fluorodeoxyglucose (^18^F-FDG), which is based upon 511 KeV gamma emissions following positron annihilation, remains a significant challenge for surgeons [1, 2]. With increasing experience in ^18^F-FDG-directed surgery techniques [1–45], multiple investigators have recognized the challenges related to the intraoperative *in situ* identification presumed abnormal ^18^F-FDG-avid tissue sites during radioguided oncologic surgery [2, 8, 14, 18, 19, 21, 28, 46–49].

The most significant challenge related to successful intraoperative *in situ* identification of presumed abnormal ^18^F-FDG-avid tissue sites during ^18^F-FDG-directed surgery is the particular situation of encountering a resultant low target-to-background ratio from the radiation emissions of ^18^F-FDG [2, 8, 14, 18, 19, 21, 28, 46–49]. It has been suggested by some authors that a minimum *in situ* target-to-background ratio of 1.5-to-1 for ^18^F-FDG is necessary for allowing the surgeon to comfortably differentiate tumor-bearing tissues from that of normal tissue during ^18^F-FDG-directed surgery [14, 18, 19]. However, this target-to-background ratio represents an arbitrary and fixed ratio determination that can be affected by multiple factors, including ^18^F-FDG uptake by tumor-bearing tissues, the distribution and degree of background radiation within various surrounding tissues which do not represent tumor-bearing tissues, and innumerable factors related to the technical specifications of the specific detection probe system used for making counts per second measurements. Our own experience with ^18^F-FDG-directed surgery [1–5, 20, 22–27, 29, 31, 41] has shown us that the observed *in situ* target-to-background ratio of presumed abnormal ^18^F-FDG-avid tissue is commonly less than 1.5-to-1, and as previously stated is highly dependent upon the specific detection probe system [2]. As a result, when intraoperative detection of *in situ*^18^F-FDG-avid tissue sites relies solely on a fixed target-to-background ratio (i.e., ratiometric threshold method) as the threshold for probe positivity, the success of such detection methods can be limited and resultantly frustrating for the surgeon [2]. Therefore, we have suggested that improved intraoperative *in situ* identification of ^18^F-FDG-avid tissue sites can be accomplished by the use of the three-sigma statistical threshold criteria method for determination of gamma detection probe positivity [2].

The three-sigma statistical threshold criteria for determination of gamma detection probe positivity has been previously well characterized by Thurston [2, 50–53], and has been previously well-utilized for radioimmunoguided surgery involving ^125^I-labeled anti-TAG-72 monoclonal antibodies [2, 50–58]. The three-sigma statistical threshold criteria defines any given tissue as being probe positive when the count rate in that tissue exceeds three standard deviations above the mean count rate detected within normal adjacent tissue [2, 50–58].

To further investigate our contention that improved intraoperative *in situ* identification of ^18^F-FDG-avid tissue sites can be accomplished by the use of the three-sigma statistical threshold criteria method for determination of gamma detection probe positivity, we recently evaluated the success rate of intraoperative *in situ* detection of presumed abnormal ^18^F-FDG-avid tissue sites using the 1.5-to-1 ratiometric threshold criteria method and the three-sigma statistical threshold criteria method for three different gamma detection probe systems in a limited data set [2]. This limited data set consisted of a group of seven patients, representing a total of nine separate ^18^F-FDG-avid tissue sites, in which all ^18^F-FDG-avid tissue sites were identified by same-day preoperative diagnostic positron emission tomography/computed tomography (PET/CT) imaging, were intraoperatively assessed *in situ* with all three gamma detection probe systems, and were subsequently surgically excised. In this analysis, we found that successful intraoperative *in situ* detection of ^18^F-FDG-avid tissue sites was more frequently accomplished by using the three-sigma statistical threshold criteria method than by using the 1.5-to-1 ratiometric threshold criteria method with each of the three gamma detection probe systems tested. Nevertheless, due to the small sample size of our limited data set that was available for the 2 × 3 contingency table analysis, there was no significant difference in our statistical analysis of overall comparison of the three gamma detection probe systems utilized as a function of the specific threshold criteria method for the determination of gamma detection probe positivity [2].

In the current analysis, and in order to rectify the above problem faced in our previous report regarding limited sample size availability for statistical analysis [2], we purposefully chose to examine the effects of utilizing the 1.5-to-1 ratiometric threshold criteria method versus the three-sigma statistical threshold criteria method for determination of gamma detection probe positivity for intraoperative *in situ* identification of presumed abnormal ^18^F-FDG-avid tissue sites within our entire study population in a manner that was completely independent of the specific type of gamma detection probe system used. As a result, this particular approach allowed for a much larger number of intraoperative gamma detection probe measurement sets that were available for the statistical analysis.

## Methods

### Logistics for deriving the complete data set from individual intraoperative gamma detection probe measurement sets of *in situ*counts

The data analyzed herein were obtained from the master database of a Cancer IRB-approved, prospective, pilot study protocol (approved by The Ohio State University Cancer IRB) involving patients undergoing ^18^F-FDG-directed surgery for known or suspected malignancy at the Arthur G. James Cancer Hospital and Richard J. Solove Research Institute of The Ohio State University Wexner Medical Center. Sixty-five patients originally gave informed consent to participate in the Cancer IRB-approved, prospective, pilot study protocol, of which 60 patients were taken to the operating room, of which 58 patients underwent ^18^F-FDG-directed surgery using available gamma detection probes, and of which 52 patients underwent appropriate *in situ* evaluation of presumed abnormal ^18^F-FDG-avid tissue sites (i.e., measurements taken before any such tissue was surgically excised) using various combinations of 6 different available gamma detection probe systems. Thus, 6 of the 58 patients who underwent ^18^F-FDG-directed surgery using available gamma detection probes did not have *in situ* evaluation of presumed abnormal ^18^F-FDG-avid tissue sites, and those 6 patients only underwent *ex situ* evaluation of presumed abnormal ^18^F-FDG-avid tissue sites (i.e., measurements taken after any such tissue was surgically excised). Additionally, none of the *in situ* gamma detection probe measurement sets in the 52 patients undergoing appropriate *in situ* evaluation of presumed abnormal ^18^F-FDG-avid tissue sites using various combinations of 6 different available gamma detection probe systems were excluded from the analysis.

Ninety-seven separate sites were selected based on identification of finite areas of presumed abnormal ^18^F-FDG-avidity seen on a preoperative diagnostic ^18^F-FDG PET/CT scan from the 52 patients described who eventually underwent appropriate *in situ* evaluation of presumed abnormal ^18^F-FDG-avid tissue sites. All presumed abnormal ^18^F-FDG-avid tissue sites were defined as abnormal as based upon the reading and official radiology report issued on the preoperative diagnostic ^18^F-FDG PET/CT scan by the reporting attending nuclear medicine physician and were not defined as abnormal as based upon any specific predetermined cut-off level for the standardized uptake value (SUV) seen in those ^18^F-FDG-avid tissue sites. Intraoperatively, these 97 *in situ* presumed abnormal ^18^F-FDG-avid sites were evaluated with various combinations of 6 available gamma detection probe systems in these 52 patients. As a result, 401 intraoperative gamma detection probe measurement sets of *in situ* counts per second measurements were cumulatively taken from among the 6 available gamma detection probe systems, representing the complete data set used in our current statistical analyses.

These 6 available gamma detection probe systems included 4 commercially-available gamma detection probe systems and 2 prototype experimental probe designs. The 4 commercially-available gamma detection probe systems consisted of 2 commercially-available gamma detection probe systems which were originally designed to detect lower energy gamma-emitting radioisotopes (Neoprobe® 1000 and Neoprobe® 2000, formally Neoprobe Corporation, Dublin; Ohio) and 2 commercially-available gamma detection probe systems which were specifically designed in an attempt to detect 511 KeV high-energy gamma emissions from ^18^F-FDG positron annihilations (Neoprobe® High Energy Probe, formally Neoprobe Corporation, Dublin; Ohio; and RMD Navigator GPS™ Gamma-PET™ Probe, formally RMD Instruments, Watertown, Massachusetts). The 2 prototype experimental probe designs consisted of: (1) a modified version of a lower energy gamma-emitting radioisotopes-detecting commercially-available probe, retro-fitted with a tungsten collimator of sufficient thickness to block 90% of 511 KeV gamma radiation, and which was intended to limit the field-of-view of the detection probe in order to increase the target-to-background ratio; and (2) an experimental detection probe which incorporated an x-ray fluorescence element to transfer the 511 KeV high-energy gamma radiation to the K-alpha x-ray energy of the element (i.e., 72 KeV), which allowed for this lower K-alpha x-ray energy to be counted at a higher efficiency than the 511 KeV high-energy gamma emissions [2, 59].

The entire group of 401 intraoperative gamma detection probe measurement sets of *in situ* counts per second measurements collected from our entire study population were cumulatively analyzed in a manner that was completely independent of the specific type of gamma detection probe system used to evaluate gamma detection probe positivity for intraoperative *in situ* identification of presumed abnormal ^18^F-FDG-avid tissue sites by the 1.5-to-1 ratiometric threshold criteria method and by the three-sigma statistical threshold criteria method.

### Definition of each intraoperative gamma detection probe measurement sets of *in situ*counts

As previously reported [2], for each intraoperative gamma detection probe measurement set, an averaged count rate (i.e., counts per second) was taken from an area selected for the *in situ* measurement of background tissue count rate, and from the area of presumed abnormal ^18^F-FDG-avid tissue selected for the *in situ* measurement of target tissue count rate. An area of presumed normal tissue within a region adjacent to the area of the target tissue was selected for the measurement of background tissue count rate. Three separate recorded values were used to generate each averaged target tissue count rate measurement determined for each area of presumed abnormal ^18^F-FDG-avid tissue. All values used for the averaged count rate measurements were normalized and reported as averaged counts per second. Count rate normalization consists of dividing acquired counts by the count duration in seconds. It is important to emphasize that all of the averaged target tissue count rate measurements that were reported in this paper represented measurements taken on an area of presumed abnormal ^18^F-FDG-avid tissue before any such tissue was surgically excised (i.e., *in situ* measurements). None of the averaged target tissue count rate measurements that were reported in this paper represented measurements taken on an area of presumed abnormal ^18^F-FDG-avid tissue after any such tissue was surgically excised (i.e., *ex situ* measurements).

### Rationale for recording *in situ*target tissue count rate measurements for the intraoperative identification of presumed abnormal ^18^F-FDG-avid tissue sites

A major inherent limitation in performing PET/CT oncologic imaging with ^18^F-FDG is related to the fact that ^18^F-FDG is not a cancer-specific imaging agent, and for which ^18^F-FDG readily accumulates within tissues representing benign disease processes (i.e., infection, inflammation, and trauma) and ^18^F-FDG readily accumulates within various normal tissues (i.e., brain, heart, mucosa and smooth muscle of the stomach, small intestines and colon, thyroid, liver, spleen, kidneys, ureters, and bladder) that have a typical physiologic propensity for ^18^F-FDG accumulation [1, 2]. These sites of benign disease accumulation and physiologic accumulation of ^18^F-FDG can result in an intrinsically high level of background ^18^F-FDG activity within regions of presumed normal tissues that may co-exist with presumed abnormal ^18^F-FDG-avid tissue sites. This can be particularly challenging when any given presumed abnormal ^18^F-FDG-avid tissue site has a relatively low target site count rate. Resultantly, recording the *in situ* measurements for the averaged target tissue count rate on any given area of presumed abnormal ^18^F-FDG-avid tissue before any such tissue was surgically excised would represent the worst-case scenario as related to the intrinsic background activity of ^18^F-FDG. Conversely, recording the *ex situ* measurements for the averaged target tissue count rate on any given area of presumed abnormal ^18^F-FDG-avid tissue after any such tissue was surgically excised would represent the best-case scenario as related to the intrinsic background activity of ^18^F-FDG. Therefore, to most definitively determine whether there was any significant difference between these two threshold detection criteria methodologies (i.e., the 1.5-to-1 ratiometric threshold criteria method and the three-sigma statistical threshold criteria method) for determination of probe positivity during attempted identification of presumed abnormal ^18^F-FDG-avid tissue sites during radioguided oncologic surgery, we selected the worst-case scenario as related to the intrinsic background ^18^F-FDG activity and elected to record the *in situ* measurements for the averaged target tissue count rate on all presumed abnormal ^18^F-FDG-avid tissue site before any such tissue sites were surgically excised, in order to maximize the negative impact of the intrinsic background ^18^F-FDG activity.

### Definition and determination of the 1.5-to-1 target-to-background ratio method for probe positivity

A target-to-background ratio was calculated for each target tissue as defined as the ratio of the averaged target tissue count rate to the background tissue count rate. The ratiometric threshold of 1.5-to-1 or greater was set as the ratiometric threshold criteria for probe positivity (i.e., 1.5-to-1 ratiometric threshold criteria method).

### Definition and theoretical derivation of the three-sigma statistical threshold criteria method of probe positivity

For each intraoperative target tissue measurement, a three-sigma statistical threshold criteria count rate was calculated by the methodology popularized of Thurston [2, 50–53]. The three-sigma statistical threshold criteria was determined from an area of presumed normal background tissue by multiplying the standard deviation of the normal background count rate in an area of presumed normal background tissue by a factor of three, and then adding that calculated number to the mean value of the normal background count rate. The condition for probe positivity was met if the count rate for the target tissue (i.e., target count rate) exceeded the calculated three-sigma criteria count rate threshold.

The three-sigma statistical threshold criteria represents a specific application of binary hypothesis testing. The theoretical deviation of the three-sigma statistical threshold criteria as it relates to radioactivity has its origins in the classical work of Currie [60]. In the classical application to radioactivity by Currie [60], the presence of a radiation source is tested against the null hypothesis that no source is present in excess of the background radiation. The resulting threshold for the alternative hypothesis, i.e. that there is a source present, can be reduced to a single variable, *μ*_*B*_, the mean value of the background radiation count. This threshold for accepting the alternative hypothesis *H*_1_ takes the form:
1

where *K*_1_ and *K*_2_ are constants. For counts rates exceeding 30 per second, *K*_1_ is considered negligibly small and the threshold for accepting the alternative hypothesis is
2

In the current application, the presumed ^18^F-FDG-avid tissue site contains activity from both background radiation and the source radiation. To derive the preceding relationship, the combined count rates must be taken into account. Although the following derivation is applied to raw counts, the same analysis can be applied to count rate measurements with no loss of generality if the count rates are calculated using equal time intervals of raw count measurements, and provided that the raw counts are sufficient to support the statistical analysis without introducing error secondary to insufficient sample size.

Radioactive decay is a Poisson distributed discrete random process since each event is independent of the preceding event. If the number of counts in a Poisson distribution exceeds approximately 30, a continuous Gaussian distribution provides a close approximation [61]. However, the value for standard deviation used in the Gaussian approximation must be equal to the square root of the mean value since this is a characteristic of the original Poisson distribution. If this condition is imposed, the two distributions can be used interchangeably and Z-score statistics can be applied using the one-sided Gaussian approximation. Counts are discrete values in both the Poisson and the corresponding Gaussian approximation.

**Figure 1:**
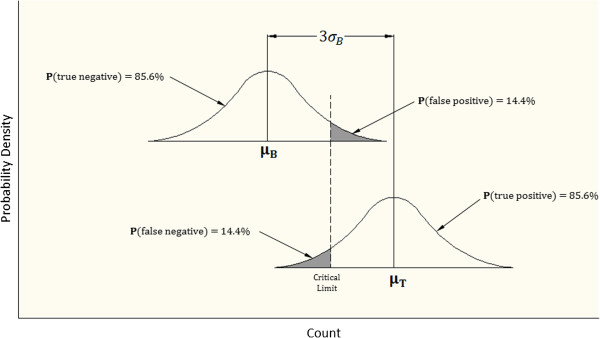
**Illustration of the probability distribution for the background count measurement and the target count measurement.** The target count measurement (***T***) represents the summation of source count measurement (***S***) plus the background count measurement (***B***). Probabilities (***P***) for true positive, true negative, false positive, and false negative are shown for target count rates at three standard deviations above the mean background count.

Consider the two probability distributions of counts illustrated in Figure 1, with one count distribution representing the background count measurement, and with the second count distribution representing the target count measurement. By definition, the target count measurement (***T***) is the summation of source count measurement (***S***) plus the background count measurement (***B***). Since the source count is the difference between the target count and background count, the variance of the source count is the sum of the variances of the target count and the background count.
3

When no source activity is present, the variance for the target is equal to the variance for the background and:
4

The Critical Limit (L_C_) for false positive counts is based on the one-sided Z parameter. A value of K is chosen for the desired percentile of statistical certainty above the mean background count. K times the standard deviation of the source count distribution defines the L_C_ at the desired percentile such that:
56

If the same K parameter is chosen to define an equal percentile of false negative and false positive probabilities, the L_C_ will also coincide with:
78

where *μ*_*D*_ is the desired source count corresponding to the minimum detectable count difference.

The standard deviation for the minimum detectible distribution, *σ*_*D*_, can also be expressed as a function of the standard deviation for both the background count and target count. Substituting the square root mean count values for the standard deviations reduces the following expression.
9

Since *μ*_*D*_ = *L*_*C*_ + *Kσ*_*D*_, the L_C_ as a function of the mean background count can be substituted.
10

This equation can be algebraically reduced to:
11

*K*^2^ (a Z-table value) is less than 3 for all certainty levels less than 95.6%, and can be considered negligible for mean background counts greater than 30. This reduces the minimum detectable count threshold to:
12

Calculating the percentile for the three-sigma statistical threshold criteria:
1314

The one-sided percentile calculation for a Z-score of 1.0607 is approximately equal to 85.6% [62].Thus, for the three-sigma statistical threshold criteria, the probability for either a false positive or false negative finding is approximately 14.4%. The confidence level for probe positivity is 85.6%. Comparing the distribution of the background count and the target count in Figure 1 illustrates the relationship between these probabilities. The probabilities of false positive and false negative findings are calculated as the area under the curve in the shaded regions indicated in Figure 1.

### Statistical analysis

**Figure 2:**
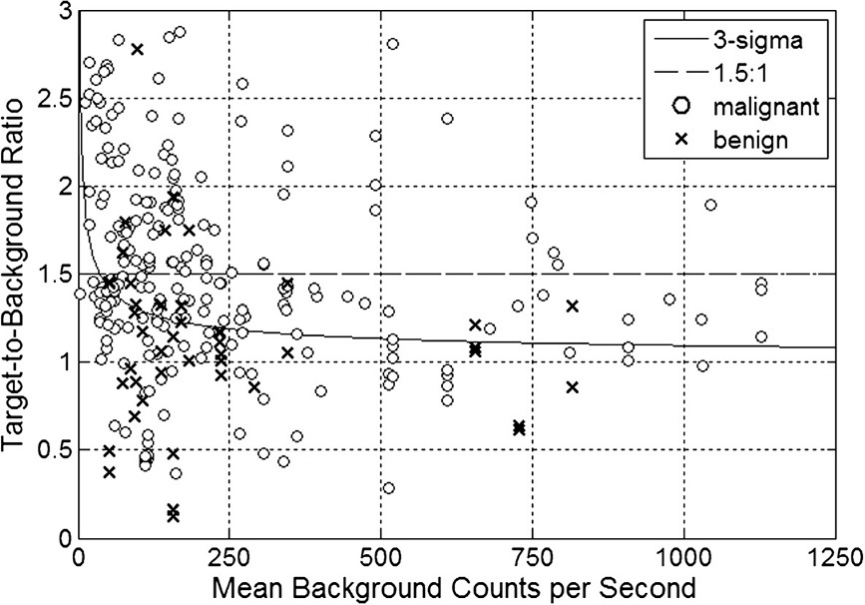
**Plot of the target-to-background ratio and mean background count rate for those 291 of the 401 gamma detection probe measurement data sets that were limited to a mean background count rate range of less than 1250 counts per second.** Malignant ^18^F-FDG-avid tissue sites are shown as (***o***) and benign^18^F-FDG-avid tissue sites are shown as (***x***). The thresholds for probe positivity, expressed as target-to-background ratios, are graphed as a function of the mean background count rate. The ratiometric threshold criteria of 1.5-to-1 is shown as a dashed line and the three-sigma threshold criteria is shown as a solid line curve.

**Figure 3:**
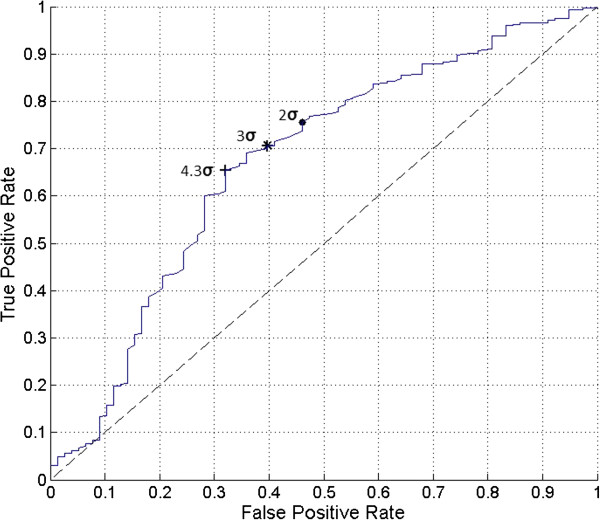
**The receiver operating characteristic (ROC) curve for various statistical threshold criteria values for probe positivity from the entire group of 401 gamma detection probe measurement sets is illustrated.** The data curve is labeled with the specific numbers of standard deviations indicated. The dashed line indicates a hypothesis test of no diagnostic discrimination. The ROC curve ranges from −46 to 74 standard deviations above the mean background count rate. The operating point on the data curve for three standard deviations above the mean background count rate is indicated as three-sigma threshold criteria (*), and corresponds to a true positive rate of 71% and false positive rate of 40%. Two-sigma operating point is indicated by (●). The optimal operating point is indicated by (+). The area under the ROC curve is 0.6728 (67.3%).

**Figure 4:**
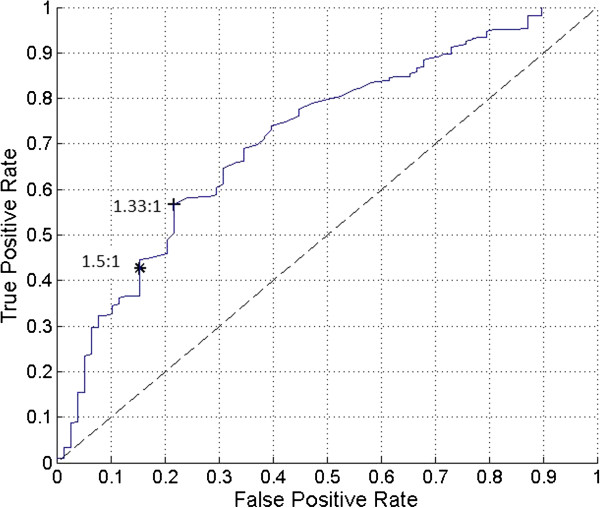
**The receiver operating characteristic (ROC) curve for various ratiometric threshold criteria values for probe positivity from the entire group of 401 gamma detection probe measurement sets is illustrated.** The ROC curve ranges from target-to-background ratios of −1-to-1 to 9-to-1 above the mean background count rate. The 1.5-to-1 ratiometric operating point is indicated by (*). The 1.33-to-1 (optimal) ratiometric operating point is indicated by (+). The area under the ROC curve is 0.7150 (71.5%).

The software program IBM SPSS® 21 for Windows® (SPSS, Inc., Chicago, Illinois) was used for all data analyses. All continuous variable results were expressed as mean (±SD, range). The probe positivity comparison for the 1.5-to-1 ratiometric threshold criteria method versus the three-sigma statistical threshold criteria method was made by way of a 2 × 2 contingency table analysis using the Pearson chi-square test. The P-value for the Pearson chi-square test comparison was reported as an exact two-sided P-value. A P-value of 0.05 or less was considered to be statistically significant.The drawing software program AutoCAD LT 2010 (AutoDesk, Inc., San Rafael, California) was used for the generation of Figure 1.The software program MATLAB R2013b version 8.2.0.701 (MathWorks, Natick, Massachusetts) was used for the generation of Figures 2, 3, and 4.

## Results and discussion

Of the 52 patients undergoing *in situ* evaluation of presumed abnormal ^18^F-FDG-avid tissue sites using any given gamma detection probe system at the time of ^18^F-FDG-directed surgery, there were 30 Caucasian females, 18 Caucasian males, 2 African-American female, 1 African-American male, and 1 Asian female. The mean patient age was 58 (±10, range 37–83) years. The mean patient body weight was 81.7 (±20.2, range 43.5-142.9) kilograms. The mean patient same-day pre-scanning blood sugar level of 106 (±18, range 78–157) milligrams per deciliter. The mean ^18^F-FDG injection dose was 537 (±55, range 387–611) megabecquerels. The mean time from ^18^F-FDG injection to the time of the start of surgery was 242 (±82, range 108–449) minutes. The mean time from ^18^F-FDG injection to the time of the start of appropriate *in situ* evaluation of presumed abnormal ^18^F-FDG-avid tissue sites using any of the given gamma detection probe system was 278 (±88, range 145–501) minutes.

The mean of the averaged target tissue count rate for the 401 gamma detection probe measurement sets was 2,047 (±3,706, range 6–16,353) counts per second. The mean of the background tissue count rate in an area of presumed normal tissue within a region adjacent to the presumed ^18^F-FDG-avid tissue site for the 401 gamma detection probe measurement sets was 1,709 (±3,172, range 4–13,020) counts per second. The mean of the calculated 1.5-to-1 target-to-background ratio for the 401 gamma detection probe measurement sets was 1.53 (±0.93, range 0.12-9.88). The mean of the calculated three-sigma statistical threshold criteria count rate for the 401 gamma detection probe measurement sets was 1,796 (±3,257, range 11–13,362) counts per second.

For the 401 gamma detection probe measurement sets, probe positivity was successfully met by the 1.5-to-1 ratiometric threshold criteria method in 150/401 instances (37.4%) and by the three-sigma statistical threshold criteria method in 259/401 instances (64.6%) (P < 0.001).

Our current statistical analysis, which intentionally utilized a very large number of individual gamma detection probe measurement sets (n = 401) of presumed abnormal ^18^F-FDG-avid tissue sites that were evaluated completely independent of the count rate determinations by any specific type of gamma detection probe system, clearly demonstrates that the three-sigma statistical threshold criteria method was significantly better than the 1.5-to-1 ratiometric threshold criteria method for determination of gamma detection probe positivity for intraoperative *in situ* identification of presumed abnormal ^18^F-FDG-avid tissue sites during ^18^F-FDG-directed surgery.

The authors fully acknowledge and accept the well-established fact that the finding of ^18^F-FDG-avidity within any given area of tissue is not an absolute indication that one will find the presence of malignancy within that given area of tissue. To further explore this subject matter, subset analysis was subsequently performed, as specifically based upon the postoperative histopathologic determination of the finding of malignant tissue versus benign tissue from all tissue specimens comprising the entire group of 401 gamma detection probe measurements sets initially analyzed. Table 1 shows the arbitrary breakdown of *in situ* probe positivity and *in situ* probe negativity versus the postoperative histopathologic determination of the finding of malignant tissue versus benign tissue. Of the 401 gamma detection probe measurements sets taken *in situ* from presumed abnormal ^18^F-FDG-avid tissue sites, postoperative histopathologic evaluation identified 323 malignant tissue specimens and 78 benign tissue specimens. Figure 2 shows the plot of the target-to-background ratio and mean background count rate for each malignant tissue site (o) and each benign tissue site (x) for those 291 of the 401 gamma detection probe measurement data points within the area of relevancy (i.e. around the ratiometric threshold criteria range of 1.5-to-1 for the target-to-background ratio and within the lower background count rate range of less than 1250 counts per second). Table 2 shows the frequency of true positives (TP), false positives (FP), true negatives (TN), and false negative (FN) versus threshold criteria method. Table 2 illustrates the increase in the number of malignant tissue sites identified as *in situ* probe positive using the three-sigma statistical threshold criteria method as compared to the 1.5-to-1 ratiometric threshold criteria method. As evident in Figure 2, there were numerous *in situ* probe positive measurement data points above the solid line curve for the three-sigma statistical threshold criteria curve and below the 1.5-to-1 ratiometric threshold criteria dashed line.The solid line in Figure 2, representing the three-sigma statistical threshold criteria curve, illustrates the advantage of the three-sigma statistical threshold criteria over that of the 1.5-to-1 ratiometric threshold criteria. Unlike the ratiometric threshold, in theory, the three-sigma statistical threshold approaches a 1-to-1 target-to-background ratio as the mean background count rate increases. For this reason, a gamma detection probe of higher counting efficiency is capable of detecting lower target-to-background ratios when the three-sigma statistical threshold criteria is applied. At increased mean background count rates, the three-sigma statistical threshold criteria can theoretically detect target-to-background ratios as low as 1.1-to-1 with a statistical confidence level of greater than 85.6%, as previously shown in Figure 1 (i.e., probability of true positives) and in the derivation of three-sigma statistical threshold criteria method of probe positivity in the Methods section.

**Table 1:** **Definitions for** ***in situ*** **probe positivity and** ***in situ*** **probe negativity versus the postoperative histopathologic determination of the finding of malignant tissue versus benign tissue**

Postoperative histopathologic determination	***in situ***probe positive	***in situ***probe negative
Malignant	True Positive (TP)	False Negative (FN)
Benign	False Positive (FP)	True Negative (TN)

**Table 2:** **Frequency of true positives (TP), false positives (FP), true negatives (TN), and false negative (FN) versus threshold criteria method**

1.5-to-1 ratiometric threshold criteria method				Three-sigma statistical threshold criteria method			
TP	FP	TN	FN	TP	FP	TN	FN
138	12	66	185	228	31	47	95

By contrast, in theory, the ratiometric threshold criteria method for determining probe positivity will result in an inconsistent statistical confidence level for target count rate measurements. The statistical confidence level of target count rate measurements is reduced at lower target count rates. As a result, the statistical confidence level for probe positivity when applying the ratiometric threshold criteria is an unknown variable at the time of *in situ* target count rate measurement. This is not the case when using statistical threshold criteria methods for probe positivity, since the statistical confidence level remains constant regardless of the count rate of the individual target count rate measurements.

The sensitivity (i.e., true positive rate) and the specificity (i.e., true negative rate) for the two threshold criteria methods (i.e., 1.5-to-1 ratiometric threshold criteria and three-sigma statistical threshold criteria) are shown in Table 3, and were calculated for the entire group of 401 gamma detection probe measurement sets using the postoperative histopathologic determination of malignant tissue versus benign tissue from Table 1 and the frequency of true positives (TP), false positives (FP), true negatives (TN), and false negative (FN) versus threshold criteria method from Table 2. The use of the three-sigma statistical threshold criteria method resulted in a 28% increase in sensitivity and a 25% decrease in specificity. It is our contention that increased sensitivity of a detection probe system generally represents a much more influential variable for affecting long-term patient outcome than does the loss of specificity of a detection probe system. The advantage of the higher sensitivity is the allowance of fewer instances in which areas of malignant tissue will go undetected and inadvertently unresected within any given patient. The negative impact of a lower specificity (i.e., more false positive findings) is the increased frequency of possible unnecessary surgical excision of areas of nonmalignant tissue in any given patient. From a clinical perspective, inadvertently leaving unresected malignant tissues within any given patient represents a far greater risk to the patient than does surgically excising tissues from the patient that are later found to be nonmalignant. This premise is well-supported by long-term survival data from antigen-directed cancer surgery for patients with primary colorectal cancer [63, 64]. For this reason, the three-sigma statistical threshold criteria method represents a marked improvement over the 1.5-to-1 ratiometric threshold criteria method for the successful intraoperative detection of malignant tissue.

**Table 3:** **Sensitivity and specificity calculations for each threshold criteria method**

Statistical metric	1.5-to-1 ratiometric threshold criteria method	Three-sigma statistical threshold criteria method
Sensitivity TP/(TP + FN)	0.43	0.71
Specificity TN/(TN + FP)	0.85	0.60

In generally, the receiver operating characteristic (ROC) is a concept used in signal detection theory to illustrate the performance of a binary classification system as the discrimination threshold is varied [65–67]. In the current analysis, the ROC provides an alternative method for comparing true positive rate and false positive rate (1-specificity). Each point on any given ROC curve is the true positive rate (sensitivity) plotted against the false positive rate (1-specificity) resulting from the threshold for probe positivity calculated for a single value. At each new threshold for probe positivity, the sensitivity and specificity were recalculated. It is essential to understand that the ROC curve, as well as sensitivity and specificity, are measuring the predictive values of not only the threshold for probe positivity, but also the entire diagnostic system, including the specifications of the gamma detection probe system utilized, as well as the subjective selection by the surgeon of the background site and the target tissue sites for recording each measurement data set. Moreover, differing data sets result in some change to the ROC curve. However, within a single ROC curve, examining the effect of changing the threshold on sensitivity and specificity is valid, provided that the changes in the statistical confidence level for the analysis is also taken into consideration.

The ROC curve for various statistical threshold criteria values for probe positivity from the entire group of 401 gamma detection probe measurement sets is illustrated in Figure 3. Figure 3 illustrates the ROC of the current data set as the number of standard deviations was varied from a value of −46 to 74 in nonlinear increments to generate a complete curve. The ROC curve was generated by varying the number of standard deviations that must be exceeded above the mean background count rate for a probe positive threshold. The operating point on the data curve for three standard deviations above the mean background count rate (i.e., three-sigma statistical threshold) indicated by the * symbol in Figure 3, corresponds to a true positive rate of 71% and false positive rate of 40%. Ideally, the true positive rate of the diagnostic test would be maximized (close to 100%) and the false positive rate would be minimized (approaching 0%) [65]. The current data set suggested that a statistical threshold using fewer than three standard deviations above the mean background count rate can increase the true positive rate without exceeding a 50% false positive rate. However, this modification would also result in a reduction in the statistical confidence level of the measurement. A statistical threshold for probe positivity of the mean background count rate plus two standard deviations, for example, would reduce the statistical confidence level of a true positive and true negative measurement from a 85.6% probability for three-sigma statistical threshold to a 76.0% probability for the for two-sigma statistical threshold.

It is also a common practice to identify the point on the ROC curve that represents the best combination of specificity and sensitivity [67]. In Figure 3, this point occurs at the location on the data curve that is closest to a true positive rate of a value of one and a false positive rate of a value of zero, which is located in the upper left hand corner of the plot. For the current data set shown in Figure 3, the best combination of specificity and sensitivity, as indicated by the + symbol, occurred at a true positive rate of 65% and false positive rate of 32%, and corresponds to a probe positive threshold level of the mean background count rate plus 4.3 standard deviations. Although utilizing this approach for identifying an optimal operating point on the ROC curve is commonplace, the resultant sensitivity of 65% for any such diagnostic instrument which would be used for detecting ^18^F-FDG-avid tissue sites would be considered suboptimal and would make any such diagnostic instrument clinically ineffective. Therefore, it is our opinion that the three-sigma statistical threshold criteria (i.e., probe positive threshold level of the mean background count rate plus 3 standard deviations) for determination of gamma detection probe positivity represents a good balance of sensitivity, selectivity, and statistical confidence levels for positive and negative probabilities. This contention for our support of the three-sigma statistical threshold criteria for determination of gamma detection probe positivity is further borne out in the fact that the three-sigma statistical threshold criteria is routinely used in other medical applications and commercial products that detect signals in the presence of background noise, such as radar detectors [68–70].

The ROC curve for various ratiometric threshold criteria values for probe positivity from the entire group of 401 gamma detection probe measurement sets is illustrated in Figure 4. A similar analysis can be applied to the ratiometric threshold for probe positivity. Figure 4 illustrates the ROC of the current data set as the ratio for target-to-background count rate for probe positivity is varied from −1-to-1 to 9-to-1. The optimal operating point the ratiometric threshold ROC curve occurred at a target-to-background ratio of 1.33-to-1. It should be noted that this value validates our earlier contention that a target-to-background ratio that is lower than 1.5-to-1 is required to adequately detect ^18^FDG-avid sites which are subsequently determined to be malignant.

In Table 4, we have summarized the frequency of true positives (TP), false positives (FP), true negatives (TN), and false negative (FN) as a function of four various probe positive threshold criteria, including (1) three-sigma statistical threshold (i.e., mean background count rate plus 3 standard deviations; (2) 4.3-sigma statistical threshold (i.e., mean background count rate plus 4.3 standard deviations; (3) target-to-background count rate ratio of 1.5-to-1 ratiometric threshold; and (4) target-to-background count rate ratio of 1.33-to-1 ratiometric threshold. The 1.33-to-1 ratiometric threshold represented a marked improvement over the 1.5-to-1 ratiometric threshold as the detection of true positives was increased from 138 to 183, and the frequency of false negative measurements was reduced from 185 to 140. Given the impact to patient outcome, improvements in these two parameters were more significant than the corresponding reduction in the frequency of true negatives and increase in the frequency of false positive measurements. Neither of the ratiometric threshold criteria values for probe positivity was as accurate in the correct detection of true positive and false negative sites as was either of the statistical threshold criteria values. Moreover, the three-sigma statistical threshold criteria method provides the best outcome for detection of both true positive and false negative sites.

**Table 4:** **Frequency of true positives (TP), false positives (FP), true negatives (TN), and false negative (FN) as a function of four various probe positive threshold criteria**

Threshold criteria method	TP	FP	TN	FN
3-Sigma Statistical	228	31	47	95
4.3-Sigma Statistical	211	25	53	112
1.5-to-1 Ratiometric	138	12	66	185
1.33-to-1 Ratiometric	183	17	61	140

## Conclusion

Our current data analysis demonstrates that the three-sigma statistical threshold criteria method is significantly better than the 1.5-to-1 ratiometric threshold criteria method for determination of gamma detection probe positivity for intraoperative *in situ* detection of presumed abnormal ^18^F-FDG-avid tissue sites during radioguided oncologic surgery. Moreover, the three-sigma statistical threshold criteria method can detect true positive results at target-to-background ratios that are much lower than the 1.5-to-1 target-to-background ratio of the 1.5-to-1 ratiometric threshold criteria method. If a surgeon utilizes a gamma detection probe system with high count rate sensitivity, it is theoretically feasible that target-to-background ratios as low as 1.1-to-1 can be identified as *in situ* probe positive ^18^F-FDG-avid tissue sites when applying the three-sigma statistical threshold criteria method. This finding may be extremely important for reshaping the ongoing and future research and development of gamma detection probe systems that are necessary for optimizing the *in situ* detection of radioisotopes of higher-energy gamma photon emissions used during radioguided oncologic surgery.
